# Multimodality salvage treatment incorporating ivonescimab plus chemotherapy for EGFR-mutated NSCLC with leptomeningeal metastases after dose-escalated third-generation EGFR-TKI failure: a retrospective study

**DOI:** 10.3389/fimmu.2026.1973923

**Published:** 2026-10-05

**Authors:** Qichao Wu, Lei Qiu, Juan An, Weiran Xu, Xiaosheng Ding, Hui Shi, Yichun Hua, Xixi Zheng, Yijia Guo, Beihe Cao, Xiaoyan Li

**Affiliations:** Department of Oncology, Beijing Tiantan Hospital, Capital Medical University, Beijing, China

**Keywords:** dose-escalated third-generation EGFR-TKI, EGFR-mutated non-small cell lung cancer, ivonescimab, leptomeningeal metastases, salvage therapy

## Abstract

**Background:**

Leptomeningeal metastasis (LM) is among the most devastating modes of progression in EGFR-mutated non-small cell lung cancer (NSCLC). Although dose escalation of third-generation EGFR-TKIs has been an important central nervous system (CNS)-directed salvage strategy, therapeutic options after progression on this intensification approach remain limited.

**Methods:**

This single-center retrospective study included consecutive patients treated between September 2024 and December 2025. Eligible patients were adults (≥18 years) with histologically confirmed lung adenocarcinoma, a documented EGFR mutation, and LM diagnosed by gadolinium-enhanced MRI or positive cerebrospinal fluid (CSF) cytology. Patients received ivonescimab plus chemotherapy after documented intracranial or extracranial progression on a dose-escalated third-generation EGFR-TKI regimen used for LM management, with or without concurrent CSF-directed therapy. Ivonescimab was given intravenously at 20 mg/kg every 3 weeks with chemotherapy selected according to prior treatment and clinical status. The primary endpoints were overall survival (OS) and LM progression-free survival (LM-PFS).

**Results:**

Of 517 screened patients, 33 initiated ivonescimab plus chemotherapy and comprised the overall treated cohort. Concurrent intrathecal or intraventricular chemotherapy was administered to 29 of 33 patients (87.9%). The median age was 61 years (IQR 56–62); 24 (72.7%) were female; 24 (72.7%) had baseline ECOG performance status of 2–3. The median number of prior therapy lines was 4 (IQR 3–6). At a median follow-up of 8.28 months, median OS was 8.54 months (95% CI 5.75–14.32), median LM-PFS was 6.54 months (95% CI 3.42–11.86), and median systemic PFS was 4.83 months (95% CI 2.79–7.56). Three-month treatment persistence was 64.4% (95% CI 44.9–78.6). Among 29 patients evaluable for LM response, 6 achieved a response, yielding an LM response rate of 20.7% (95% CI 8.0–39.7) and an LM disease control rate of 82.8% (95% CI 64.2–94.2). Among 31 patients with evaluable safety documentation, grade ≥3 treatment-related adverse events occurred in 14 (45.2%), most commonly hematologic toxicities. No permanent ivonescimab discontinuation due to toxicity was documented.

**Conclusions:**

This cohort provides real-world outcome data for patients with EGFR-mutated NSCLC and LM receiving multimodality salvage treatment incorporating ivonescimab plus chemotherapy after dose-escalated third-generation EGFR-TKI failure.

## Introduction

Leptomeningeal metastasis (LM) is a particularly devastating complication of epidermal growth factor receptor (EGFR)-mutated non-small cell lung cancer (NSCLC) (1). Although advances in targeted therapy have prolonged systemic survival, they have also increased the number of patients living long enough to develop LM, making it an increasingly important clinical challenge (2–5). LM remains associated with substantial neurological morbidity, functional deterioration, and poor survival, and its diagnosis, monitoring, and treatment remain challenging in routine practice (6–12). Current evidence for LM treatment has largely centered on EGFR-tyrosine kinase inhibitor (TKI) intensification (4, 13–18). Dose-escalated or otherwise CNS-directed third-generation EGFR-TKIs are commonly used as an initial salvage approach. BLOOM, BLOSSOM, and other studies of high-dose third-generation EGFR-TKIs have documented activity in selected patients with LM (19, 20). However, the optimal systemic treatment after failure of dose-escalated third-generation EGFR-TKI remains uncertain.

This post-intensification setting is clinically important but poorly studied. Prospective trials specifically addressing LM remain uncommon because of the relatively low incidence of LM and the difficulties associated with trial enrollment. Moreover, patients with LM have often been excluded from broader therapeutic trials, underrepresented in enrolled populations, or included without separate LM-specific efficacy outcomes. Although chemotherapy-based, antiangiogenic, and antibody-based approaches can retain antitumor activity after EGFR-TKI resistance, their efficacy in LM, particularly after failure of intensified third-generation EGFR-TKI, remains unclear.

Recently, the retrospective FURMO-LM study found that furmonertinib-based triplet therapy comprising an antiangiogenic agent and chemotherapy was associated with longer OS than mono or dual therapy in EGFR-mutated NSCLC patients with LM, including those with prior third-generation TKI resistance (21). Its findings support the potential value of incorporating chemotherapy and vascular endothelial growth factor (VEGF) pathway inhibition into treatment strategies for TKI-resistant LM. Ivonescimab, a bispecific antibody targeting programmed cell death protein 1 (PD-1) and VEGF, in combination with chemotherapy has shown antitumor activity in patients with EGFR-TKI-treated NSCLC, including those with brain metastases, providing a rationale for further evaluation in later-line disease (22, 23). Whether this strategy has clinically relevant activity against LM is unknown. We therefore evaluated outcomes of multimodality salvage treatment incorporating ivonescimab plus chemotherapy in patients with EGFR-mutated NSCLC and LM whose disease had progressed after dose-escalated third-generation EGFR-TKI therapy.

## Materials and methods

### Study design and patient selection

This single-center, retrospective cohort study was conducted at the Department of Oncology, Beijing Tiantan Hospital, and included consecutive eligible patients treated between September 2024 and December 2025. The data cutoff date was March 31, 2026. The source population was identified by retrospectively reviewing institutional LM records dating back to 2021.

Patients were eligible if they were 18 years of age or older, had histologically or cytologically confirmed lung adenocarcinoma with a documented EGFR mutation identified by real-time polymerase chain reaction or next-generation sequencing, and had LM diagnosed by gadolinium-enhanced magnetic resonance imaging (MRI) and/or positive cerebrospinal fluid (CSF) cytology (6, 24). All patients had also received ivonescimab plus chemotherapy after documented intracranial or extracranial progression on a dose-escalated third-generation EGFR-TKI regimen used for LM management, including osimertinib 160 mg daily or furmonertinib 160 mg daily. The prior dose-escalated third-generation EGFR-TKI could have been administered with or without concurrent intrathecal or intraventricular chemotherapy. Patients were excluded if available records were insufficient to confirm eligibility, treatment initiation, or survival follow-up.

This cohort comprised patients with LM requiring salvage treatment after failure of an LM-directed dose-escalated third-generation EGFR-TKI strategy. Failure of the preceding strategy could be documented by LM progression, progression at non-LM sites with persistent clinically active LM, or combined LM and other-site progression.

### Treatment regimens

Ivonescimab was administered intravenously at 20 mg/kg every 3 weeks until disease progression, unacceptable toxicity, or treatment discontinuation at the treating physician’s discretion, and was combined with chemotherapy on a 3-week cycle. The chemotherapy backbone was selected on the basis of prior treatment exposure, disease status, and overall clinical condition, and consisted mainly of pemetrexed- or taxane-based regimens, with or without platinum, or single-agent chemotherapy in selected patients.

Concurrent CSF-directed therapy consisted primarily of intrathecal or intraventricular pemetrexed or methotrexate (25–30). The decision to use concurrent intrathecal or intraventricular chemotherapy was based on neurological findings, CSF cytology, prior LM-directed treatment, and patient preference. Treatment was withheld upon clearance of malignant cells on serial CSF cytology together with stabilization of CNS symptoms.

### Assessments

Baseline clinical characteristics, disease characteristics, previous anticancer treatments, EGFR-TKI exposure, LM characteristics, CSF cytology, neurological status, and concomitant CSF-directed treatment were collected from medical records. Eastern Cooperative Oncology Group (ECOG) performance status was recorded at baseline and during treatment when available. 

Radiographic assessments included contrast-enhanced brain MRI and computed tomography (CT) of the chest, abdomen, and pelvis, performed at baseline and approximately every 2 cycles thereafter. LM response was assessed retrospectively using the 2017 RANO-LM framework, integrating contrast-enhanced MRI, neurological findings, and CSF cytology obtained during routine care (31). Imaging assessment included linear and nodular leptomeningeal enhancement, encompassing measurable and nonmeasurable disease. Outcomes were classified as response, stable disease (SD), or progressive disease (PD) based on the composite assessment. LM response referred to the composite category in **Supplementary eTable 4** of the 2017 RANO-LM proposal. It required neurological improvement, definite MRI improvement in leptomeningeal disease, and negative CSF cytology, with no progression in any domain. In this cohort, patients without baseline leptomeningeal enhancement on brain MRI were classified as SD or PD when sufficient follow-up was available. LM progression was defined by unequivocal radiographic worsening or new LM lesions, conversion of CSF cytology from negative to positive, or unequivocal deterioration on neurological examination attributable to LM. Parenchymal brain metastases were assessed separately using RANO-BM criteria (32). Extracranial disease was assessed separately using RECIST version 1.1 (33).

Assessments were performed by one independent medical oncologist with expertise in neuro-oncology, blinded to the original response assessments documented in the medical records and subsequent clinical outcomes. The formal RANO-LM neurological scorecard was not used; neurological changes were assessed qualitatively from contemporaneous clinical records using the definitions in eMethod 2. CSF samples were obtained by lumbar puncture or via an Ommaya reservoir when clinically indicated, particularly during concurrent CSF-directed therapy, to monitor cytologic status over time.

Safety was assessed retrospectively from physician notes, laboratory results, and treatment-modification records. The safety analysis included events documented as treatment-related, graded according to the Common Terminology Criteria for Adverse Events (CTCAE), version 5.0. Treatment-related dose reductions and permanent discontinuations were summarized separately.

### End points

The primary endpoints were OS and LM-PFS. Secondary endpoints included systemic PFS, LM response and disease control, extracranial objective response rate (ORR) and disease control rate (DCR), treatment persistence, descriptive ECOG change, and safety.

OS was defined as the interval from initiation of ivonescimab plus chemotherapy to death from any cause. Systemic PFS was defined as the interval from treatment initiation to the earliest documented disease progression at any site or death from any cause, whichever occurred first. LM-PFS was defined as the interval from treatment initiation to LM progression based on the RANO-LM assessment or death from any cause, whichever occurred first. All three time-to-event endpoints were assessed in the overall treated cohort (n = 33), including patients without a post-baseline response assessment. Patients without an event were censored at the last relevant follow-up or disease assessment. LM response was assessed in patients with sufficient post-baseline LM follow-up (n = 29), whereas extracranial RECIST response required baseline measurable extracranial target lesions and at least one post-baseline assessment (n = 25). In sensitivity analyses, missing responses were counted as non-responses in all 33 treated patients for LM response and all 29 patients with baseline measurable extracranial disease for extracranial ORR. Endpoint-specific populations are summarized in **Supplementary eTable 3**.

Three-month treatment persistence was defined as the Kaplan–Meier–estimated probability of remaining on ivonescimab-containing treatment 3 months after treatment initiation, without permanent treatment discontinuation or death. Safety endpoints were descriptive and included treatment-related adverse events (AEs) graded according to CTCAE version 5.0 and treatment modifications attributable to toxicity. ECOG change from baseline to the best recorded status during treatment was summarized descriptively.

### Statistical analysis

Analyses were performed in R (version 4.4.0), with updated survival and response summaries calculated in Python (version 3.12). OS, LM-PFS, systemic PFS, and treatment persistence were estimated using the Kaplan–Meier method, with corresponding 95% confidence intervals (CIs). LM response and disease control rates and extracranial ORR and DCR were summarized descriptively in the corresponding endpoint-specific evaluable populations, with 95% CIs calculated using the Clopper–Pearson exact method. Safety analyses were descriptive. Treatment exposure and treatment modifications were summarized in the overall treated cohort. Treatment-related AE incidence was summarized among patients with retrievable post-treatment safety records.

No formal sample-size calculation was performed because this was a retrospective, descriptive real-world cohort study in a narrowly defined and rare post-intensification LM salvage setting. The study size was determined by the number of consecutive eligible patients in the institutional lung adenocarcinoma LM registry who met all prespecified eligibility criteria and initiated ivonescimab plus chemotherapy during the study period. Median follow-up was calculated using the reverse Kaplan–Meier method. No imputation was performed for missing baseline or safety variables. Missing response assessments were handled as specified in the sensitivity analyses. Available-case analyses were used, with denominators reported for each endpoint-specific analysis population.

## Results

### Patient characteristics

Of the 517 patients in the lung adenocarcinoma LM registry, 175 had documented progression on dose-escalated third-generation EGFR-TKI therapy. Of these, 33 (18.9%) initiated ivonescimab plus chemotherapy at our center (**Figure 1**). The remaining 142 patients (81.1%) did not receive this regimen at our center. Their subsequent treatment outside our center and individual reasons for non-initiation were not systematically ascertained. Baseline characteristics are summarized in **Table 1** and **Supplementary eTable 1**. All 33 patients were included in the OS, LM-PFS, and systemic PFS analyses. LM response was assessed in 29 patients (87.9%), extracranial RECIST response in 25 (75.8%), and safety in 31 (93.9%).

**Table 1:** Baseline characteristics of the included cohort.

Characteristic	Overall (N = 33)
Assessment sets and evaluability	
Included cohort, n	33
LM response-evaluable population, n (%)	29 (87.9)
Measurable extracranial RECIST population, n (%)	25 (75.8)
AE-evaluable records, n (%)	31 (93.9)
Demographics	
Age, years	61 (56–62)
Sex, n (%)	
Female	24 (72.7)
Male	9 (27.3)
Smoking history, n (%)	
Yes	6 (18.2)
No	27 (81.8)
ECOG performance status, n (%)	
1	9 (27.3)
2	16 (48.5)
3	8 (24.2)
LM diagnosis and CNS disease burden	
LM diagnostic evidence, n (%)	
Cytology-confirmed	31 (93.9)
Non-cytology diagnosis (MRI-based)	2 (6.1)
MRI leptomeningeal/cranial nerve enhancement before ivonescimab initiation, n (%)	25 (75.8)
Baseline brain metastases, n (%)	
Present	29 (87.9)
Absent	4 (12.1)
Number of parenchymal brain metastases among BM-positive patients, n/N (%)	
Single (1 lesion)	3/29 (10.3)
Multiple (≥2 lesions)	26/29 (89.7)
Molecular characteristics	
Primary EGFR mutation, n (%)	
Exon 19 deletion	13 (39.4)
L858R	20 (60.6)
Prior treatment burden	
Lines of therapy from initial treatment to ivonescimab, median (IQR)	4 (3–6)
≥4 lines from initial treatment to ivonescimab, n/N (%)	22/33 (66.7)
Prior chemotherapy, n (%)	22 (66.7)
Prior immunotherapy, n (%)	7 (21.2)
Prior antiangiogenic therapy, n (%)	21 (63.6)
Prior brain radiotherapy, n (%)	17 (51.5)
Time from LM diagnosis to ivonescimab, months, median (IQR)	8.7 (6.9–10.9)
PFS on prior LM-directed dose-escalated third-generation EGFR-TKI, months, median (IQR)	4.0 (3.0–9.0)
Progression at prior EGFR-TKI failure, n (%)	
LM only	11 (33.3)
LM and other sites	10 (30.3)
Non-LM sites only	12 (36.4)

Data are presented as n (%) unless otherwise indicated; continuous variables are summarized as median (IQR).

BM, brain metastases; CNS, central nervous system; ECOG, Eastern Cooperative Oncology Group; EGFR, epidermal growth factor receptor; EGFR-TKI, epidermal growth factor receptor tyrosine kinase inhibitor; IQR, interquartile range; LM, leptomeningeal metastasis; MRI, magnetic resonance imaging; PFS, progression-free survival.

**Figure 1:**
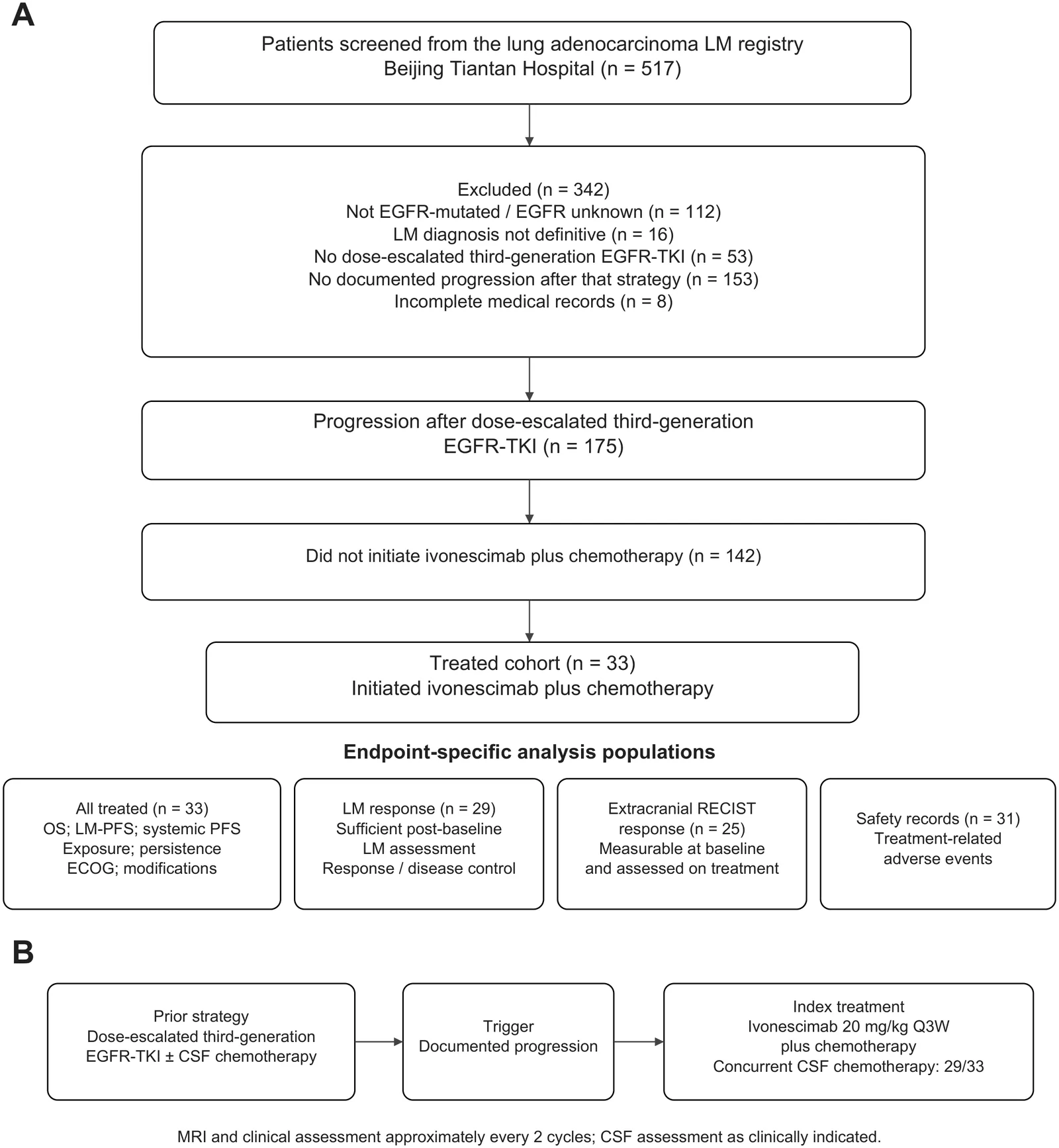
Patient selection, post-intensification salvage treatment schema, and endpoint-specific analysis populations. **(A)** Patient screening and endpoint-specific analysis populations. Of 517 patients screened, 175 had documented progression after dose-escalated third-generation EGFR-TKI, and 33 initiated ivonescimab plus chemotherapy. The remaining 142 patients did not receive the study regimen at our center. Their subsequent treatment outside our center and individual reasons for non-initiation were not systematically ascertained. The overall treated cohort was used for OS, LM-PFS, systemic PFS, treatment exposure, treatment persistence, descriptive ECOG shift, and treatment-modification analyses. LM response was assessed in 29 patients with sufficient post-baseline follow-up, extracranial RECIST response in 25 patients with baseline measurable disease and post-baseline assessment, and treatment-related AEs in 31 patients with evaluable safety documentation. **(B)** Prior LM-directed strategy, progression trigger, and index treatment. Concurrent intrathecal or intraventricular chemotherapy was administered to 29 patients. Disease assessment was performed approximately every 2 cycles, with CSF evaluation as clinically indicated. CSF, cerebrospinal fluid; LM, leptomeningeal metastasis; OS, overall survival; PFS, progression-free survival; Q3W, every 3 weeks.

This cohort was heavily pretreated with a median age of 61 years (IQR, 56–62); 24 patients (72.7%) were female, 27 (81.8%) were never-smokers, and 24 (72.7%) had a baseline ECOG performance status of 2–3. LM was cytology-confirmed in 31 patients (93.9%); baseline brain MRI before ivonescimab initiation showed leptomeningeal or cranial nerve enhancement in 25 patients (75.8%). Neurological symptoms were documented in all 33 patients before initiation of ivonescimab plus chemotherapy (**Supplementary eTable 1**). Baseline parenchymal brain metastases were present in 29 patients (87.9%), with multiple lesions in 26 of 29 (89.7%). EGFR mutations comprised exon 19 deletion in 13 patients (39.4%) and L858R in 20 (60.6%). The median number of lines of therapy from initial treatment to ivonescimab was 4 (IQR, 3–6); 22 patients (66.7%) had received at least 4 lines, and prior exposure was frequent for chemotherapy (66.7%), antiangiogenic therapy (63.6%), immunotherapy (21.2%), and brain radiotherapy (51.5%). The median time from LM diagnosis to ivonescimab initiation was 8.7 months (IQR, 6.9–10.9), and the median PFS on the prior LM-directed dose-escalated third-generation EGFR-TKI was 4.0 months (IQR, 3.0–9.0). Detailed exposure to the prior LM-directed dose-escalated third-generation EGFR-TKI and corresponding failure patterns are summarized in **Supplementary eTable 2**. At data cutoff (March 31, 2026), the median follow-up was 8.28 months. At failure of the preceding EGFR-TKI regimen, 11 patients (33.3%) had LM-only progression, 10 (30.3%) had LM progression with progression at other sites, and 12 (36.4%) had progression confined to non-LM sites.

### Treatment exposure

Treatment exposure is summarized in **Table 2**, Section A. The chemotherapy backbone was predominantly pemetrexed plus platinum, administered to 23 patients (69.7%); the remaining patients received taxane plus platinum [3 (9.1%)], taxane monotherapy [4 (12.1%)], pemetrexed monotherapy [2 (6.1%)], or a mixed/sequential regimen [1 (3.0%)]. Concurrent intrathecal or intraventricular chemotherapy was administered to 29 patients (87.9%): 22 (75.9%) received pemetrexed alone, 4 (13.8%) methotrexate alone, and 3 (10.3%) both agents. The median number of administrations was 2 (IQR, 1–4; range, 0–9) in the overall cohort and 2 (IQR, 1–4; range, 1–9) among those receiving concurrent treatment. Median cumulative doses administered at our center during ivonescimab plus chemotherapy were 80 mg (range, 20–360) for pemetrexed (n = 25) and 20 mg (range, 10–60) for methotrexate (n = 7). Among the 4 patients without concurrent CSF-directed treatment, three had become CSF cytology-negative after prior dose-escalated third-generation EGFR-TKI plus CSF-directed chemotherapy, and one declined further CSF-directed treatment. No patient received brain radiotherapy during ivonescimab plus chemotherapy. In exploratory descriptive summaries, median LM-PFS was 6.54 months in the 29 patients receiving concurrent CSF-directed treatment and 1.02 months in the four without it; corresponding median OS was 8.54 and 10.97 months.

**Table 2:** Treatment exposure and key efficacy outcomes.

Characteristic	Overall (N = 33)
Section A. Treatment exposure	
Chemotherapy regimen category, n (%)	
Pemetrexed plus platinum doublet	23 (69.7)
Taxane plus platinum doublet	3 (9.1)
Taxane monotherapy	4 (12.1)
Pemetrexed monotherapy	2 (6.1)
Mixed/sequential regimen	1 (3.0)
Concurrent intrathecal or intraventricular chemotherapy actually administered, n (%)	29 (87.9)
Agent category for intrathecal or intraventricular chemotherapy among treated patients, n/N (%)	
Pemetrexed only	22/29 (75.9)
Methotrexate only	4/29 (13.8)
Both pemetrexed and methotrexate	3/29 (10.3)
Section B. Tumor response and key efficacy outcomes	
Extracranial RECIST objective response rate, n/N (%)	6/25 (24.0); 95% CI, 9.4–45.1
Extracranial RECIST disease control rate, n/N (%)	23/25 (92.0); 95% CI, 74.0–99.0
LM response rate by RANO-LM, n/N (%)	6/29 (20.7); 95% CI, 8.0–39.7
LM disease control rate by RANO-LM, n/N (%)	24/29 (82.8); 95% CI, 64.2–94.2
Section C. Time-to-event and descriptive ECOG outcomes	
Overall survival, months, median (95% CI)	8.54 (5.75–14.32)
Systemic PFS events/censored, n	21/12
Systemic PFS, months, median (95% CI)	4.83 (2.79–7.56)
LM-PFS in all treated patients, months, median (95% CI)	6.54 (3.42–11.86)
3-month treatment persistence, % (95% CI)	64.4 (44.9–78.6)
Best ECOG performance status shift during treatment, n (%)	
Improved (best < pretreatment)	11 (33.3)
Stable (best = pretreatment)	19 (57.6)
Worsened (best > pretreatment)	3 (9.1)

Data are presented as n (%) unless otherwise indicated; continuous variables are summarized as median (IQR; range) or median (95% CI). Extracranial RECIST response required baseline measurable extracranial target lesions and at least one post-baseline assessment (n = 25). LM response required sufficient post-baseline LM follow-up (n = 29). OS, LM-PFS, systemic PFS, treatment exposure, persistence, descriptive ECOG shift, and treatment modifications were assessed in all 33 treated patients.

### Efficacy

At data cutoff, 14 of 33 patients had died and 19 were censored. Kaplan–Meier curves for OS, LM-PFS, and systemic PFS are shown in **Figure 2**. In the overall treated cohort, median OS was 8.54 months (95% CI, 5.75–14.32), median LM-PFS was 6.54 months (95% CI, 3.42–11.86; 18 events and 15 censored observations), and median systemic PFS was 4.83 months (95% CI, 2.79–7.56; 21 events and 12 censored observations).

**Figure 2:**
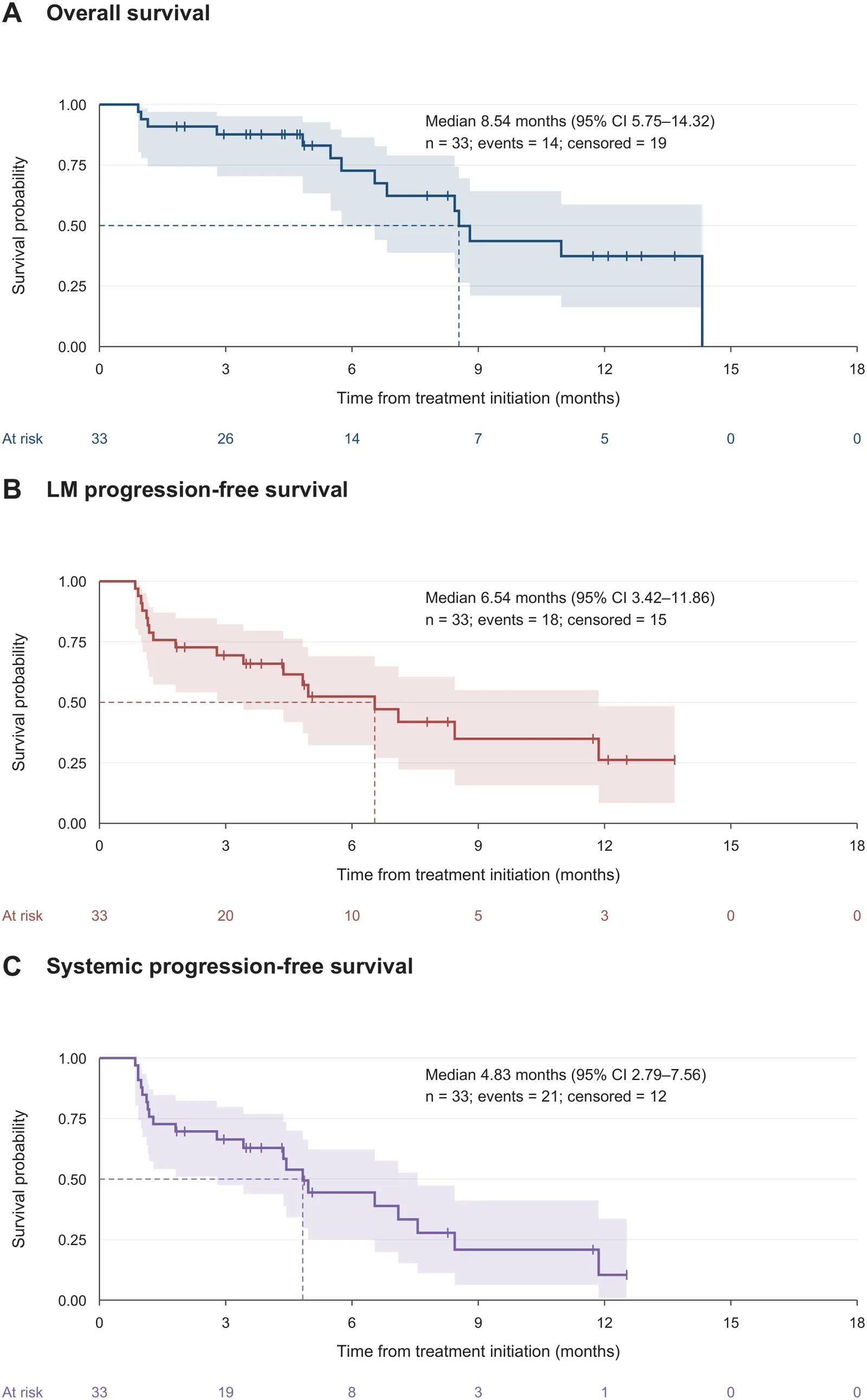
Kaplan–Meier estimates of overall survival, LM progression-free survival, and systemic progression-free survival. **(A)** OS, **(B)** LM-PFS, and **(C)** systemic PFS in the overall treated cohort (n = 33). LM-PFS includes all treated patients, including those without a post-baseline LM response assessment. Shaded areas indicate 95% confidence intervals. Tick marks denote censoring. Dashed lines indicate the median survival estimate. Numbers below each plot show patients at risk. CI, confidence interval; OS, overall survival; PFS, progression-free survival; RANO-LM, Response Assessment in Neuro-Oncology Leptomeningeal Metastases.

Individual time-to-event and response profiles are shown in **Figure 3A**, and response outcomes in **Table 2** and **Figures 3B, C**. Among 25 patients with baseline measurable extracranial target lesions and at least one post-baseline RECIST assessment, no complete responses were observed; 6 patients (24.0%) had a partial response, 17 (68.0%) had stable disease, and 2 (8.0%) had progressive disease, yielding an extracranial ORR of 24.0% (95% CI, 9.4–45.1) and a DCR of 92.0% (95% CI, 74.0–99.0). Four patients had no baseline measurable extracranial target lesions, and four lacked a post-baseline target-lesion assessment. Among 29 patients evaluable for LM response, 6 (20.7%) achieved a response, 18 (62.1%) had stable disease, and 5 (17.2%) had progressive disease. The LM response rate was 20.7% (95% CI, 8.0–39.7), and the LM disease control rate was 82.8% (95% CI, 64.2–94.2). Insufficient post-baseline response assessment reflected early treatment discontinuation or clinical progression, including death before reassessment. All 33 patients were retained in the time-to-event analyses. Counting missing assessments as non-responses yielded an LM response rate of 18.2% (6/33; 95% CI, 7.0–35.5) and an extracranial ORR of 20.7% (6/29; 95% CI, 8.0–39.7) among all patients with baseline measurable disease. Three-month treatment persistence was 64.4% (95% CI, 44.9–78.6).

**Figure 3:**
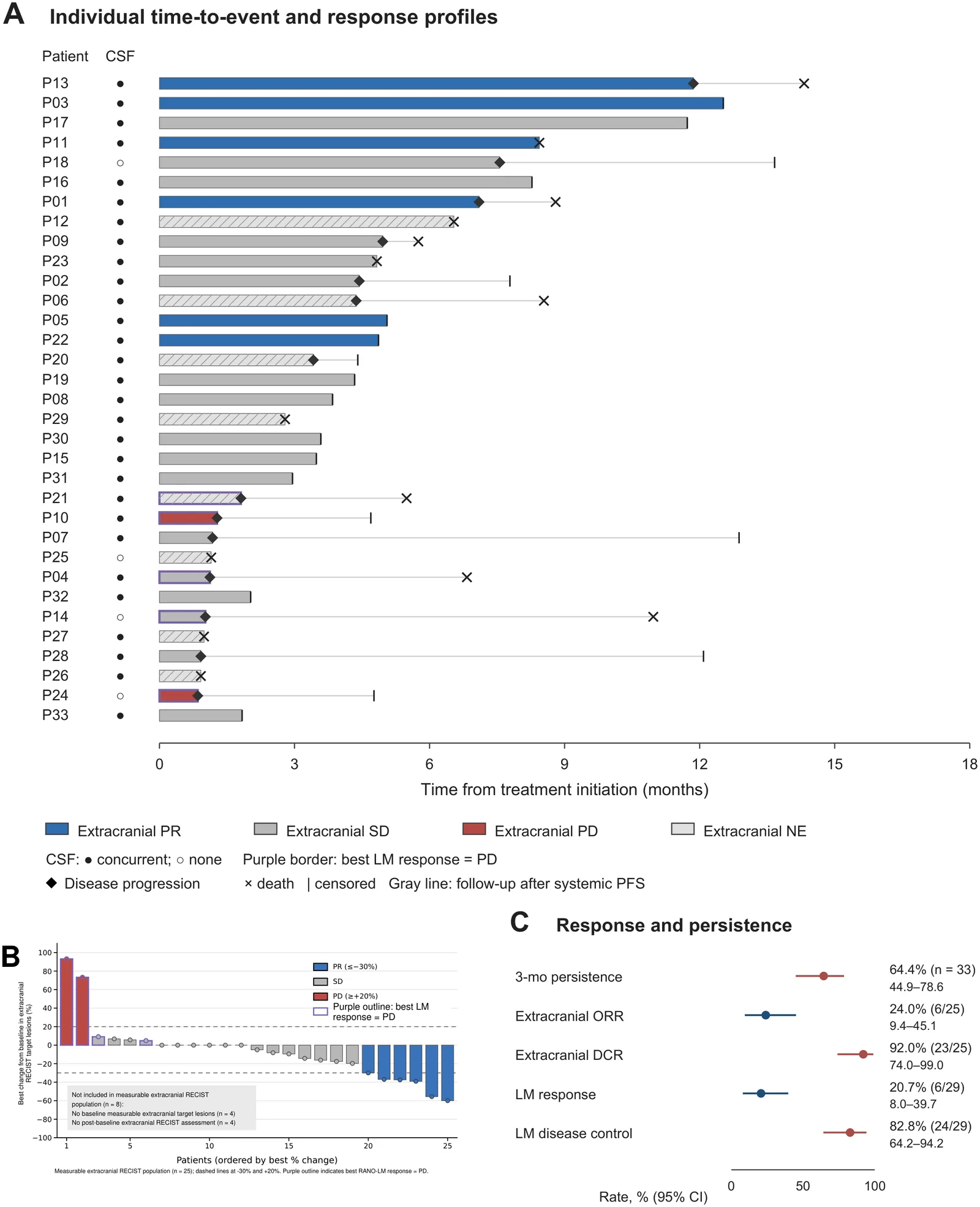
Individual time-to-event profiles and response outcomes. **(A)** Individual time-to-event profiles from initiation of ivonescimab plus chemotherapy. Colored bars extend to systemic progression, death, or censoring; thin gray lines show subsequent follow-up to death or censoring. Bar colors indicate best extracranial RECIST response, and hatching indicates non-evaluable response. The CSF column identifies concurrent intrathecal or intraventricular chemotherapy. Purple borders indicate best LM response classified as PD by RANO-LM. Diamonds denote disease progression, crosses denote death, and vertical ticks denote censoring. **(B)** Best percentage change in measurable extracranial RECIST target lesions among 25 evaluable patients. Thresholds are −30% for partial response and +20% for progressive disease. Four patients without baseline measurable lesions and four without a post-baseline target-lesion assessment are omitted. **(C)** Three-month treatment persistence (n = 33), extracranial ORR and DCR (n = 25), and LM response and disease control rates (n = 29). Response-rate CIs use the Clopper–Pearson exact method; persistence uses Kaplan–Meier estimation with Greenwood log-log CIs. CI, confidence interval; DCR, disease control rate; KM, Kaplan–Meier; NE, not evaluable; ORR, objective response rate; PD, progressive disease; PR, partial response; RANO-LM, Response Assessment in Neuro-Oncology Leptomeningeal Metastases; RECIST, Response Evaluation Criteria in Solid Tumors; SD, stable disease. CSF, cerebrospinal fluid; LM, leptomeningeal metastasis.

Best ECOG performance status during treatment improved in 11 patients (33.3%), remained unchanged in 19 (57.6%), and worsened in 3 (9.1%) (**Table 2**, Section C; **Supplementary eFigure 1**). These shifts are descriptive and do not estimate a treatment effect on functional status.

In the exploratory subgroup analysis, median systemic PFS was 7.56 months in patients with prior LM-directed dose-escalated EGFR-TKI PFS ≥6 months (n = 15) and 2.79 months in those with prior PFS <6 months (n = 18; log-rank P = 0.070). The univariable hazard ratio was 0.44 (95% CI, 0.17–1.09; P = 0.077). No corresponding OS separation was observed (**Supplementary eFigure 2**).

### Safety

Overall safety findings are summarized in **Figures 4A, B**. Among 31 patients with evaluable safety documentation, 22 (71.0%) experienced at least 1 treatment-related AE, and 14 (45.2%) experienced at least 1 grade 3 or higher AE. Treatment modifications were summarized in the overall treated cohort (n = 33): ivonescimab dose reduction attributable to AEs was documented in 3 patients (9.1%), and no permanent ivonescimab discontinuation attributable to toxicity was documented. Chemotherapy dose reduction and permanent discontinuation attributable to AEs were documented in 7 patients (21.2%) and 2 patients (6.1%), respectively. Two patients had incomplete safety documentation after transfer to other hospitals for continued treatment. Long-acting granulocyte colony-stimulating factors were used for primary prevention of chemotherapy-associated neutropenia in 19 patients (57.6%). Romiplostim was used in 11 patients (33.3%) to prevent recurrent thrombocytopenia following thrombocytopenia during prior chemotherapy. The median number of treatment cycles was 2 (IQR, 1–4; range, 1–21). All-cause deaths within 30 and 90 days of treatment initiation occurred in 2 (6.1%) and 4 (12.1%) patients, respectively.

**Figure 4:**
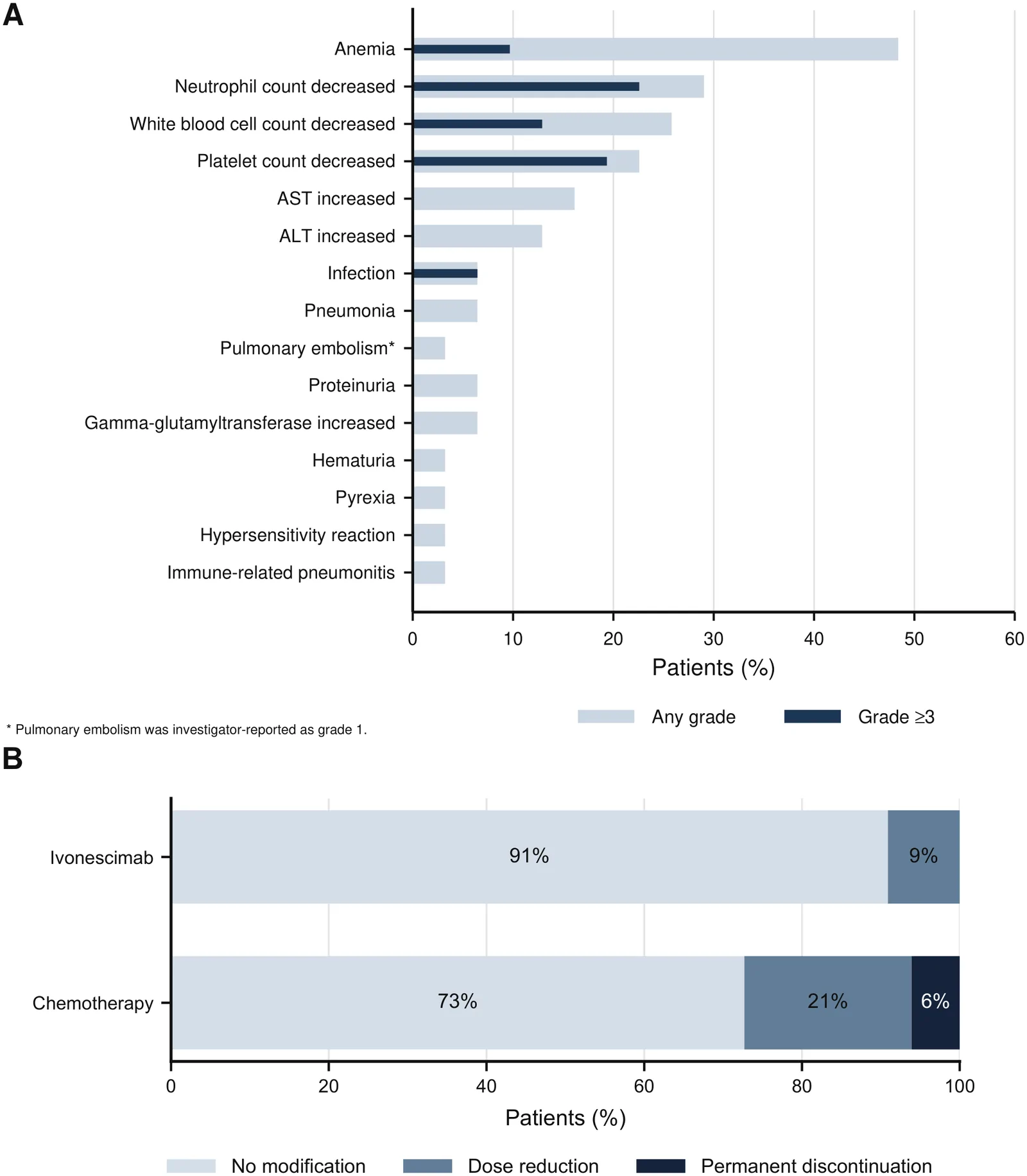
Treatment-related adverse events and treatment modifications. **(A)** Most frequent documented treatment-related adverse events among patients with evaluable post-treatment safety documentation (n = 31). Light bars indicate events of any grade, and dark bars indicate grade 3 or higher events, graded according to CTCAE version 5.0. **(B)** Treatment modifications during ivonescimab plus chemotherapy in the overall treated cohort (n = 33). Stacked bars show the proportions of patients with no modification, dose reduction, or permanent discontinuation for ivonescimab and chemotherapy, respectively. ^*^Pulmonary embolism was investigator-reported as grade 1. The event was asymptomatic and suspected on imaging but remained unconfirmed; no anticoagulation was initiated. AE, adverse event; ALT, alanine aminotransferase; AST, aspartate aminotransferase.

The toxicity profile was predominantly hematologic. The most frequently reported AEs were anemia and decreases in neutrophil, white blood cell, and platelet counts. Nonhematologic AEs were less frequent and included transaminase elevation, infection, proteinuria, pulmonary embolism, immune-related pneumonitis, increased gamma-glutamyl transferase levels, and hematuria (**Figure 4A**). A complete listing of documented treatment-related adverse events is provided in **Supplementary eTable 4**.

## Discussion

This single-center retrospective study describes LM and systemic outcomes following multimodality salvage treatment incorporating ivonescimab plus chemotherapy after dose-escalated third-generation EGFR-TKI failure. Median OS was 8.54 months, median LM-PFS was 6.54 months, and median systemic PFS was 4.83 months. Among patients evaluable for LM response, the response rate was 20.7% and the disease control rate was 82.8%.

The present findings extend the existing evidence base for EGFR-mutated NSCLC with LM beyond the setting of initial EGFR-TKI intensification. Earlier studies, including BLOOM and BLOSSOM studies, demonstrated the activity of third-generation EGFR-TKI therapy in LM. In BLOOM, osimertinib 160 mg daily produced a median PFS of 8.6 months and median OS of 11.0 months in patients with cytology-confirmed LM after prior EGFR-TKI treatment (19). AURA LM analyses reported activity with standard-dose osimertinib in EGFR T790M-positive disease (34). BLOSSOM also reported survival and intracranial activity with osimertinib 80 mg daily in patients whose LM developed after first- or second-generation EGFR-TKIs (20). Retrospective data suggest that osimertinib may improve survival regardless of T790M status, but they do not define treatment after dose-escalated third-generation EGFR-TKI failure (35). In a prospective real-world study of high-dose furmonertinib, 72.9% of 48 patients had previously received a third-generation EGFR-TKI; median OS was 8.43 months, and RANO-LM radiologic response and disease control rates were 50.0% and 92.1% (14). Other studies have evaluated intrathecal pemetrexed (36, 37). The EGFR-TKI studies support treatment intensification, but provide limited guidance once LM progresses despite intensified third-generation EGFR inhibition. An international multicenter cohort also found an association between continued third-generation EGFR-TKI use after LM diagnosis and longer survival (5). The present cohort addresses salvage treatment after failure of dose-escalated third-generation EGFR-TKI therapy.

Evidence for ivonescimab plus chemotherapy comes mainly from broader post-EGFR-TKI populations. In HARMONi-A, the combination improved PFS compared with chemotherapy alone in TKI-treated EGFR-variant NSCLC (22). MARIPOSA-2 showed that amivantamab-based chemotherapy after osimertinib progression improved systemic and intracranial PFS (38). Neither trial focused on LM or provides a direct benchmark for this cohort. A phase 2 study by Chen et al. also reported activity of amivantamab plus lazertinib in previously treated patients with EGFR-mutant NSCLC and LM (39). The TROP2-directed antibody–drug conjugate datopotamab deruxtecan has shown activity in EGFR-mutant NSCLC after targeted therapy and platinum-based chemotherapy (40). Treatment choice in this cohort depended on prior therapy, clinical status, and treatment availability.

The RANO-LM framework integrates neurological examination, CSF cytology, and neuraxis imaging for LM response assessment (31). In the present cohort, median LM-PFS was 6.54 months, compared with 4.83 months for systemic PFS. Both endpoints included all 33 treated patients; progression outside the leptomeninges contributed to systemic PFS but did not by itself constitute LM progression.

The LM response and extracranial RECIST response populations differed because some patients lacked sufficient LM follow-up, whereas others had no measurable extracranial target lesions. Reporting these analysis sets separately avoids treating extracranial response as a surrogate for LM control. LM progression was documented in 21 of 33 patients at failure of the preceding EGFR-TKI regimen, whereas 12 had progression confined to non-LM sites. LM disease-control estimates should therefore be considered alongside disease status at treatment initiation. The exploratory association between prior EGFR-TKI PFS and subsequent systemic PFS may reflect differences in disease course and does not establish a predictive treatment-selection criterion.

Twenty-nine of 33 patients received concurrent intrathecal or intraventricular chemotherapy, most commonly pemetrexed, and systemic chemotherapy backbones varied. Intrathecal or intraventricular chemotherapy is an option for selected patients with LM (2, 3). The observed outcomes therefore reflect the combined treatment strategy, and the contribution of ivonescimab cannot be separated from that of the accompanying treatments. The four patients without concurrent CSF-directed therapy provide only a small descriptive subgroup.

In our study, most patients had poor baseline functional status, with 72.7% having an ECOG performance status of 2–3, 93.9% having cytology-confirmed LM, and 87.9% having concomitant parenchymal brain metastases. Prior chemotherapy, immunotherapy, and antiangiogenic therapy had been administered to 22 (66.7%), 7 (21.2%), and 21 (63.6%) patients, respectively. These prior treatments may have influenced sensitivity to subsequent therapy and regimen selection.

Hematological toxicities were the most frequently recorded treatment-related adverse events, and grade 3 or higher events occurred in 45.2% of patients with evaluable safety documentation. Ivonescimab dose reduction because of toxicity occurred in three patients, and no permanent discontinuation because of toxicity was documented. Prophylactic hematopoietic support and retrospective ascertainment of treatment-related events may have influenced the observed event rates.

This single-center retrospective cohort is subject to selection bias and unmeasured confounding, and its small sample size limits subgroup analyses. The absence of a comparator precludes estimation of the incremental benefit of ivonescimab. Subsequent treatment outside our center and reasons for non-initiation were not systematically ascertained for the other 142 patients, limiting characterization of treatment selection. These findings apply to patients who received this salvage regimen at our center. In some patients, death occurred before further LM-specific reassessment, limiting retrospective characterization of the individual components of LM progression. Neurological changes were assessed qualitatively from retrospective clinical records; prospective studies should incorporate a standardized neurological scorecard.

Prospective LM-specific endpoints have been evaluated in a randomized trial of proton craniospinal irradiation (41). Future studies of systemic salvage treatment should standardize concurrent intrathecal or intraventricular chemotherapy, prespecify LM-specific efficacy endpoints, and use eligibility criteria that include patients with neurological impairment.

In conclusion, this study describes outcomes of multimodality salvage treatment incorporating ivonescimab plus chemotherapy in patients with EGFR-mutated NSCLC and LM after failure of dose-escalated third-generation EGFR-TKI therapy.

## Data Availability

De-identified participant data and a supporting data dictionary will be made available from the corresponding author upon reasonable request, pending institutional review and execution of a formal data use agreement. Analytic code for reproducing the primary analyses will be provided through a supplementary repository after publication. Requests to access the datasets should be directed to Xiaoyan Li, lixiaoyan@bjtth.org.
