# Supplementary material for: Multimodality salvage treatment incorporating ivonescimab plus chemotherapy for EGFR-mutated NSCLC with leptomeningeal metastases after dose-escalated third-generation EGFR-TKI failure: a retrospective study
Source: Front Immunol. 2026 Oct 5;17:1973923. doi: 10.3389/fimmu.2026.1973923 (PMC13677939; doi:10.3389/fimmu.2026.1973923)
Supplement: Supplementary file 1 [file DataSheet1.docx]

**Supplemental Data 1**

**eMethods**

eMethod 1. Eligibility criteria, LM diagnosis, and analysis-set definitions

eMethod 2. RANO-LM assessment and endpoint adjudication in routine practice

eMethod 3. Treatment exposure and concurrent intrathecal or intraventricular chemotherapy

eMethod 4. Safety assessment and statistical methods

**eTables**

eTable 1. Detailed baseline characteristics, disease context, and prior treatment history

eTable 2. Prior LM-Directed Dose-Escalated Third-Generation EGFR-TKI Exposure

eTable 3. Endpoint-specific analysis populations and outcome mapping

eTable 4. Documented treatment-related adverse events

**eFigures**

eFigure 1. Descriptive ECOG performance status changes
eFigure 2. Systemic PFS and OS stratified by prior dose-escalated EGFR-TKI PFS

**eMethods**

**eMethod 1. Eligibility criteria, LM diagnosis, and analysis-set definitions**

Patients were eligible if they met all of the following criteria: (1) age 18 years or older; (2) histologically or cytologically confirmed lung adenocarcinoma with a documented EGFR mutation identified by real-time polymerase chain reaction or next-generation sequencing; (3) a clinical diagnosis of leptomeningeal metastasis (LM) established in routine practice on the basis of gadolinium-enhanced magnetic resonance imaging (MRI), cerebrospinal fluid (CSF) cytology, or both; and (4) initiation of ivonescimab plus chemotherapy after documented intracranial or extracranial progression on a dose-escalated third-generation EGFR tyrosine kinase inhibitor (EGFR-TKI) regimen used for LM management, including osimertinib 160 mg daily or furmonertinib 160 mg daily. Prior dose-escalated third-generation EGFR-TKI treatment could have been administered with or without concurrent intrathecal or intraventricular chemotherapy. Patients were excluded if available records were insufficient to confirm eligibility, treatment initiation, or survival follow-up.

LM diagnostic evidence was summarized in the main manuscript as cytology-confirmed versus non-cytology diagnosis. More detailed diagnostic categories, including MRI plus CSF cytology and MRI-based diagnosis with supportive CSF information, are provided in eTable 1. At baseline before ivonescimab initiation, brain MRI showed leptomeningeal or cranial nerve enhancement in 25 of 33 patients (75.8%).

The overall treated cohort comprised all 33 patients who initiated ivonescimab plus chemotherapy and was used for baseline characteristics, treatment exposure, OS, LM-PFS, systemic PFS, treatment persistence, descriptive ECOG shift, and treatment-modification analyses. LM response was assessed in 29 patients with sufficient post-baseline LM follow-up. Extracranial RECIST response required baseline measurable extracranial target lesions and at least one post-baseline assessment (n = 25). Treatment-related AE incidence was summarized among 31 patients with retrievable post-treatment clinical and laboratory safety documentation. Four patients without sufficient post-baseline response assessment remained in all time-to-event analyses. Sensitivity analyses counted missing response assessments as non-responses: LM response in all 33 treated patients and extracranial ORR in all 29 patients with baseline measurable extracranial disease.

**eMethod 2. RANO-LM assessment and endpoint adjudication in routine practice**

LM response was assessed retrospectively using the 2017 RANO-LM framework, integrating contrast-enhanced MRI, neurological findings, and CSF cytology obtained during routine care. Imaging assessment included linear and nodular leptomeningeal enhancement, encompassing measurable and nonmeasurable disease. Outcomes were classified as response, stable disease (SD), or progressive disease (PD) based on the composite assessment. LM response referred to the composite category in Table 4 of the 2017 RANO-LM proposal. It required neurological improvement, definite MRI improvement in leptomeningeal disease, and negative CSF cytology, with no progression in any domain. In this cohort, patients without baseline leptomeningeal enhancement on brain MRI were classified as SD or PD when sufficient follow-up was available. LM progression was defined by unequivocal radiographic worsening or new LM lesions, conversion of CSF cytology from negative to positive, or unequivocal deterioration on neurological examination attributable to LM. Assessments were performed by one independent medical oncologist with expertise in neuro-oncology, blinded to the original response assessments documented in the medical records and subsequent clinical outcomes. Parenchymal brain metastases were assessed separately using RANO-BM criteria.

The formal RANO-LM neurological scorecard was not used. In evaluable patients, contemporaneous records supported qualitative comparisons over time. They did not consistently capture the domain-specific detail needed to reconstruct formal numerical scores reliably. Improvement required documented improvement in baseline LM-related neurological abnormalities without new or worsening LM-related findings. Deterioration required new or unequivocally worsening abnormalities attributable to LM. Alternative causes, including coexisting brain metastases, treatment toxicity, and other medical conditions, were considered before assigning this category. Stable status required sufficient longitudinal documentation showing no clinically meaningful change. Insufficient documentation was not interpreted as neurological stability.

For all LM response-evaluable patients, baseline and follow-up contrast-enhanced brain MRI examinations and CSF cytology reports were available and traceable to the original records. These findings were assessed together with documented neurological changes to determine the composite LM response. Follow-up imaging was obtained approximately every two treatment cycles. CSF reassessment was performed when clinically indicated, particularly during concurrent CSF-directed therapy. Patients without sufficient post-baseline information were considered non-evaluable for response and were retained in the all-treated sensitivity analysis.

LM-PFS was measured from treatment initiation to LM progression under the 2017 RANO-LM framework or death from any cause, whichever occurred first, in all 33 treated patients. Extracranial RECIST progression alone did not constitute LM progression. Patients without LM progression or death were censored at their last LM-relevant assessment. Death before further LM reassessment was an event rather than a reason for exclusion.

**eMethod 3. Treatment exposure and concurrent intrathecal or intraventricular chemotherapy**

Treatment exposure was summarized for all patients who initiated ivonescimab plus chemotherapy. The index date was the first administration of ivonescimab with chemotherapy after failure of an LM-directed, dose-escalated third-generation EGFR-TKI. Chemotherapy backbones, treatment duration, and subsequent modifications reflected routine practice rather than a protocol. Recorded exposure variables included the chemotherapy regimen, number of cycles, treatment persistence, and documented modification or discontinuation.

Prior LM-directed dose-escalated third-generation EGFR-TKI exposure was recorded separately to characterize the post-intensification salvage setting. These data included the agent used, dose-escalated treatment context for LM management, and relevant preceding exposure history, and are summarized in eTable 2.

Concurrent intrathecal or intraventricular chemotherapy was defined as administration during ivonescimab plus chemotherapy. The decision to use concurrent intrathecal or intraventricular chemotherapy was based on neurological findings, CSF cytology, prior LM-directed treatment, and patient preference. Administration counts and cumulative doses refer only to treatment given at our center during this period. Of the four patients without concurrent treatment, three had CSF cytology clearance after the preceding dose-escalated EGFR-TKI plus CSF-directed chemotherapy, and one declined further CSF-directed treatment. No patient received concurrent brain radiotherapy.

Outcomes reflect multimodality salvage treatment, including ivonescimab plus chemotherapy and concurrent CSF-directed treatment when administered. The subgroup without concurrent CSF-directed therapy was too small for a reliable between-group treatment-effect comparison.

**eMethod 4. Safety assessment and statistical methods**

Treatment modifications were summarized for all 33 treated patients. Treatment-related AEs were identified retrospectively from physician notes, laboratory results, and treatment-modification records and graded using CTCAE version 5.0. The event scope was treatment-related AEs documented during clinical care. Two patients had incomplete safety documentation after transfer to other hospitals for continued treatment and were excluded from AE incidence summaries, but retained in treatment exposure and time-to-event analyses. Long-acting granulocyte colony-stimulating factors were used for primary neutropenia prophylaxis in 19 patients. Romiplostim was used in 11 patients to prevent recurrent thrombocytopenia following prior chemotherapy-associated thrombocytopenia. All-cause mortality within 30 and 90 days was calculated from exact recorded treatment-start and death dates in all 33 patients; these deaths were not assumed to be treatment-related.

Analyses were performed using R (version 4.4.0), with updated survival and response summaries calculated in Python (version 3.12). Baseline characteristics, treatment exposure, and safety data were summarized descriptively. Categorical variables were reported as counts and percentages, and continuous variables were summarized as median with interquartile range, unless otherwise specified. Overall survival, systemic PFS, LM-PFS, and treatment persistence were estimated using the Kaplan–Meier method, with corresponding 95% confidence intervals. LM response and disease control rates and extracranial objective response and disease control rates were calculated with corresponding 95% confidence intervals in the prespecified evaluable populations for LM and extracranial RECIST response analyses, respectively. Because this was a descriptive retrospective real-world study, no formal between-group hypothesis testing was prespecified in the supplementary analyses. The subgroup analyses stratified by prior EGFR-TKI PFS were exploratory and post hoc. Survival distributions were compared using the log-rank test, and univariable Cox proportional hazards models were used to estimate hazard ratios and corresponding 95% confidence intervals. The prior EGFR-TKI PFS groups were <6 months (n = 18) and ≥6 months (n = 15), with <6 months as the Cox reference group. Cox models used Efron handling of tied events. Kaplan–Meier confidence intervals used the Greenwood log-log method; response-rate confidence intervals used the Clopper–Pearson exact method.

**eTable 1. Detailed baseline characteristics, disease context, and prior treatment history**

| **Characteristic** | **Overall (N = 33)** |
| --- | --- |
| **Additional LM diagnostic detail** | |
| Brain MRI plus CSF cytology, n (%) | 23 (69.7) |
| CSF cytology only, n (%) | 3 (9.1) |
| CSF cytology plus supportive CSF biomarker/molecular testing, n (%) | 5 (15.2) |
| Brain MRI only, n (%) | 1 (3.0) |
| Brain MRI plus CSF molecular testing without positive cytology, n (%) | 1 (3.0) |
| **Baseline neurologic and CSF disease context** | |
| Baseline CSF opening pressure, mmH₂O, median (IQR) | 170 (120–220) |
| Baseline CSF opening pressure >180 mmH₂O, n (%) | 16 (48.5) |
| Baseline neurologic symptoms present, n (%) | 33 (100.0) |
| Headache, n (%)¹ | 21 (63.6) |
| Dizziness/vertigo, n (%)¹ | 13 (39.4) |
| Nausea and/or vomiting, n (%)¹ | 11 (33.3) |
| Visual symptoms, n (%)¹ | 6 (18.2) |
| Cranial nerve/facial symptoms, n (%)¹ | 7 (21.2) |
| Limb weakness and/or numbness, n (%)¹ | 4 (12.1) |
| **CSF diversion/access devices history** | |
| Ventriculoperitoneal shunt present, n (%) | 10 (30.3) |
| Ommaya reservoir present, n (%) | 20 (60.6) |
| **Prior brain radiotherapy category, n (%)** | |
| None | 16 (48.5) |
| Whole-brain–containing radiotherapy | 9 (27.3) |
| Focal/stereotactic radiotherapy only | 8 (24.2) |
| Prior thoracic radiotherapy, n (%) | 5 (15.2) |
| Prior radiotherapy to other metastatic sites, n (%) | 3 (9.1) |
| **Additional molecular and treatment-burden detail** | |
| Concomitant TP53 alteration, n (%) | 15 (45.5) |
| No concomitant TP53 alteration, n (%) | 17 (51.5) |
| TP53 status unknown, n (%) | 1 (3.0) |
| Lines of therapy from LM diagnosis to ivonescimab initiation, median (IQR) | 2 (2–3) |
| 2 lines from LM diagnosis to ivonescimab, n (%) | 24 (72.7) |
| 3 lines from LM diagnosis to ivonescimab, n (%) | 8 (24.2) |
| 4 lines from LM diagnosis to ivonescimab, n (%) | 1 (3.0) |

Data are presented as n (%) unless otherwise indicated; continuous variables are summarized as median (IQR). **Abbreviations:** CSF, cerebrospinal fluid; EGFR, epidermal growth factor receptor; IQR, interquartile range; LM, leptomeningeal metastasis; MRI, magnetic resonance imaging.

*¹ Symptom categories were not mutually exclusive.*

**eTable 2. Prior LM-Directed Dose-Escalated Third-Generation EGFR-TKI Exposure**

| **Characteristic** | **Overall (N = 33)** |
| --- | --- |
| **Section A. Broader EGFR-TKI exposure history before ivonescimab** | |
| No. of distinct EGFR-TKIs received before ivonescimab, median (IQR) | 3 (2–3) |
| 1 prior EGFR-TKI | 8 (24.2) |
| 2 prior EGFR-TKIs | 8 (24.2) |
| ≥3 prior EGFR-TKIs | 17 (51.5) |
| Any prior first- or second-generation EGFR-TKI exposure | 12 (36.4) |
| Any prior osimertinib exposure | 19 (57.6) |
| Any prior furmonertinib exposure | 31 (93.9) |
| Any prior exposure to other third-generation EGFR-TKIs | 14 (42.4) |
| **Section B. Immediate pre-ivonescimab LM-directed dose-escalated third-generation EGFR-TKI regimen** | |
| Furmonertinib-based | 24 (72.7) |
| Osimertinib-based | 6 (18.2) |
| Other third-generation EGFR-TKI–based | 3 (9.1) |
| PFS on the immediate pre-ivonescimab LM-directed dose-escalated third-generation EGFR-TKI regimen, months |  |
| <6 | 18(54.5) |
| ≥6 | 15 (45.5) |
| **Section C. Documented failure pattern leading to ivonescimab initiation** | |
| Progression confined to non-LM sites | 12 (36.4) |
| LM-only progression | 11 (33.3) |
| LM progression with progression at other sites | 10 (30.3) |

Data are presented as n (%) unless otherwise indicated; continuous variables are summarized as median (IQR). Immediate pre-ivonescimab regimen categories were abstracted from recorded EGFR-TKI treatment history and categorized according to the third-generation EGFR-TKI used in the LM-directed dose-escalated regimen immediately preceding ivonescimab.

**Abbreviations:** CNS, central nervous system; EGFR, epidermal growth factor receptor; EGFR-TKI, epidermal growth factor receptor tyrosine kinase inhibitor; IQR, interquartile range; LM, leptomeningeal metastasis; PFS, progression-free survival.

**eTable 3. Endpoint-specific analysis populations and outcome mapping**

| **Analysis population** | **Definition** | **N** |
| --- | --- | --- |
| **Section A. Analysis populations and core definitions** | | |
| Overall treated cohort | Initiated ivonescimab plus chemotherapy | 33 |
| LM response-evaluable population | Sufficient post-baseline follow-up for LM response assessment | 29 |
| Measurable extracranial RECIST population | Baseline measurable extracranial target lesions and ≥1 post-baseline RECIST assessment | 25 |
| AE-evaluable records | Retrievable post-treatment clinical and laboratory safety documentation for AE grading | 31 |
| **Section B. Endpoint-to-analysis-set mapping** | | |
| **Endpoint** | **Analysis population** | |
| Baseline characteristics | Overall treated cohort | |
| Treatment exposure | Overall treated cohort | |
| OS | Overall treated cohort | |
| Systemic PFS | Overall treated cohort | |
| 3-month treatment persistence | Overall treated cohort | |
| ECOG shift | Overall treated cohort | |
| Treatment modifications | Overall treated cohort | |
| LM-PFS | Overall treated cohort (n = 33) | |
| LM response / disease control by RANO-LM | LM response-evaluable population | |
| Extracranial RECIST ORR/DCR | Measurable extracranial RECIST population | |
| AE incidence | AE-evaluable records | |

Data are presented as n (%) unless otherwise indicated. Percentages are based on the overall treated cohort (N = 33). Abbreviations: AE, adverse event; CNS, central nervous system; DCR, disease control rate; ECOG, Eastern Cooperative Oncology Group; LM, leptomeningeal metastasis; ORR, objective response rate; OS, overall survival; PFS, progression-free survival; RANO-LM, Response Assessment in Neuro-Oncology Leptomeningeal Metastases; RECIST, Response Evaluation Criteria in Solid Tumors.

**eTable 4. Documented treatment-related adverse events**

| **Characteristic** | **Any grade, n (%)** | **Grade ≥3, n (%)** |
| --- | --- | --- |
| **Section A. Safety analysis set** | | |
| Patients with evaluable post-treatment safety documentation | 31 |  |
| Patients with any adverse event | 22 (71.0) | — |
| Patients with at least 1 grade ≥3 adverse event | — | 14 (45.2) |
| **Section B. Hematologic adverse events** | | |
| Anemia | 15 (48.4) | 3 (9.7) |
| White blood cell count decreased | 8 (25.8) | 4 (12.9) |
| Neutrophil count decreased | 9 (29.0) | 7 (22.6) |
| Platelet count decreased | 7 (22.6) | 6 (19.4) |
| **Section C. Hepatic and other laboratory abnormalities** | | |
| Aspartate aminotransferase increased | 5 (16.1) | 0 (0.0) |
| Alanine aminotransferase increased | 4 (12.9) | 0 (0.0) |
| Gamma-glutamyltransferase increased | 2 (6.5) | 0 (0.0) |
| **Section D. Renal and urinary adverse events** | | |
| Proteinuria | 2 (6.5) | 0 (0.0) |
| Hematuria | 1 (3.2) | 0 (0.0) |
| **Section E. Cardiovascular, pulmonary, and infectious adverse events** | | |
| Pneumonia | 2 (6.5) | 0 (0.0) |
| Pulmonary embolism^*^ | 1 (3.2) | 0 (0.0) |
| Infection | 2 (6.5) | 2 (6.5) |
| **Section F. Neurologic, dermatologic, and general adverse events** | | |
| Pyrexia | 1 (3.2) | 0 (0.0) |
| Hypersensitivity reaction | 1 (3.2) | 0 (0.0) |
| **Section G. Immune-related adverse events** | | |
| Immune-related pneumonitis | 1 (3.2) | 0 (0.0) |

AE percentages use the 31 patients with evaluable safety documentation. Two patients with incomplete records after transfer to other hospitals remained in exposure and time-to-event analyses. The table reports documented treatment-related events; unlisted toxicities should not be interpreted as prospectively ascertained zero incidence. Treatment modifications and all-cause early mortality are summarized separately in all 33 patients. ^*^Pulmonary embolism was investigator-reported as grade 1. The event was asymptomatic and suspected on imaging but remained unconfirmed; no anticoagulation was initiated.

**Abbreviations:** AE, adverse event.

**eFigures**

**eFigure 1. Descriptive changes from baseline to best recorded ECOG performance status during treatment.**

ECOG changes are descriptive and do not estimate a treatment effect on functional status. ECOG, Eastern Cooperative Oncology Group; PS, performance status.

**eFigure 2. Systemic PFS and OS stratified by prior LM-directed dose-escalated third-generation EGFR-TKI PFS.**

(A) Systemic PFS and (B) OS in patients with prior EGFR-TKI PFS <6 months (n = 18) or ≥6 months (n = 15). Shading denotes 95% CIs; ticks denote censoring. Risk tables report patients at risk. Hazard ratios compare ≥6 versus <6 months. These post hoc analyses are exploratory. CI, confidence interval; HR, hazard ratio; NR, not reached; OS, overall survival; PFS, progression-free survival.
